# Mental Wellness Self-Care in Singapore With mindline.sg: A Tutorial on the Development of a Digital Mental Health Platform for Behavior Change

**DOI:** 10.2196/44443

**Published:** 2024-06-04

**Authors:** Janice Huiqin Weng, Yanyan Hu, Creighton Heaukulani, Clarence Tan, Julian Kuiyu Chang, Ye Sheng Phang, Priyanka Rajendram, Weng Mooi Tan, Wai Chiong Loke, Robert J T Morris

**Affiliations:** 1 MOH Office for Healthcare Transformation Singapore Singapore; 2 Yong Loo Lin School of Medicine National University of Singapore Singapore Singapore

**Keywords:** digital mental health, artificial intelligence, AI, AI chatbot, digital therapeutics, mental health, mental wellness, mobile phone

## Abstract

**Background:**

Singapore, like the rest of Asia, faces persistent challenges to mental health promotion, including stigma around unwellness and seeking treatment and a lack of trained mental health personnel. The COVID-19 pandemic, which created a surge in mental health care needs and simultaneously accelerated the adoption of digital health solutions, revealed a new opportunity to quickly scale innovative solutions in the region.

**Objective:**

In June 2020, the Singaporean government launched mindline.sg, an anonymous digital mental health resource website that has grown to include >500 curated local mental health resources, a clinically validated self-assessment tool for depression and anxiety, an artificial intelligence (AI) chatbot from Wysa designed to deliver digital therapeutic exercises, and a tailored version of the website for working adults called mindline at work. The goal of the platform is to empower Singapore residents to take charge of their own mental health and to be able to offer basic support to those around them through the ease and convenience of a barrier-free digital solution.

**Methods:**

Website use is measured through click-level data analytics captured via Google Analytics and custom application programming interfaces, which in turn drive a customized analytics infrastructure based on the open-source platforms Titanium Database and Metabase. Unique, nonbounced (users that do not immediately navigate away from the site), engaged, and return users are reported.

**Results:**

In the 2 years following launch (July 1, 2020, through June 30, 2022), the website received >447,000 visitors (approximately 15% of the target population of 3 million), 62.02% (277,727/447,783) of whom explored the site or engaged with resources (referred to as nonbounced visitors); 10.54% (29,271/277,727) of those nonbounced visitors returned. The most popular features on the platform were the dialogue-based therapeutic exercises delivered by the chatbot and the self-assessment tool, which were used by 25.54% (67,626/264,758) and 11.69% (32,469/277,727) of nonbounced visitors. On mindline at work, the rates of nonbounced visitors who engaged extensively (ie, spent ≥40 seconds exploring resources) and who returned were 51.56% (22,474/43,588) and 13.43% (5,853/43,588) over a year, respectively, compared to 30.9% (42,829/138,626) and 9.97% (13,822/138,626), respectively, on the generic mindline.sg site in the same year.

**Conclusions:**

The site has achieved desired reach and has seen a strong growth rate in the number of visitors, which required substantial and sustained digital marketing campaigns and strategic outreach partnerships. The site was careful to preserve anonymity, limiting the detail of analytics. The good levels of overall adoption encourage us to believe that mild to moderate mental health conditions and the social factors that underly them are amenable to digital interventions. While mindline.sg was primarily used in Singapore, we believe that similar solutions with local customization are widely and globally applicable.

## Introduction

### Mental Health and Wellness in Singapore

Mental health conditions are now responsible for 1 in 5 years lived with disability [1], and depression is the leading cause of disability worldwide, affecting >300 million people [2]. In Singapore, 13.9% of the adult population experienced at least 1 mood, anxiety, or alcohol use disorder in their lifetime [3]. As in other Asian countries, the treatment gap is large; 78.6% of individuals met the criteria for a 12-month mental health condition and were in need of mental health care but did not receive help or treatment [4]. Asia faces unique challenges in improving mental health. The region experiences a shortage of mental health professionals [5,6]. For example, in 2016, Singapore and South Korea reported 4.19 and 5.79 psychiatrists, respectively, working in the mental health sector per 100,000 population [7]; most Southeast Asian countries have <1 psychiatrist per 100,000 population [6]. In comparison, Australia reported 13.53 psychiatrists per 100,000 population in 2015, and the United Kingdom reported 17.98 psychiatrists per 100,000 population in 2019 [7,8].

Digital solutions for mental health are poised to overcome these challenges. Wearable devices and smartphones provide increased accessibility and less-stigmatizing avenues for mental health care [9], and there is evidence to support the use of internet-based interventions for mental health treatment [10,11] and self-help [12,13]. The COVID-19 pandemic has accelerated the adoption of these tools; there was an increase in the use of digital mental health technology during the pandemic in the United States [14], and in Singapore, there was a rise in demand for digital mental health services and mobile apps [15]. The market today is large; there are an estimated 10,000 to 20,000 mental health mobile apps on the market today [16], and mental health start-ups garnered >US $1 billion in funding in the first half of 2020 [17].

In June 2020, driven by the COVID-19 pandemic, the MOH Office for Healthcare Transformation (under the Ministry of Health in Singapore) launched a digital mental health resource website called mindline.sg. Initially intended to serve emergent mental health needs following lockdowns and major societal disruption, the initiative has evolved to address the broader gap in digital mental wellness provision in the country. In particular, there is a need to recognize that mental health exists on a spectrum and maintaining a healthy state of mental well-being involves being equipped with the necessary skills and knowledge for coping with regular life stresses [18]. To this end, *mental wellness* (or *mental well-being*) can be defined as a positive state of mental health beyond the absence of a mental health condition; being mentally well means you are able to think, feel, and act in ways that create a positive impact on your physical and social well-being [18]. Where the health care system primarily serves the needs of those diagnosed with a mental health condition, digital mental wellness self-help resources may provide a scalable complement that enables people to maintain their mental wellness.

To the best of our knowledge, mindline.sg is the first of its kind in Asia. The platform provides >500 curated local resources, a clinically validated self-assessment tool for depression and anxiety, and an emotionally intelligent artificial intelligence (AI) chatbot from Wysa delivering a suite of interactive digital therapies (the chatbot will be described in detail in the Methods section). Data-driven digital marketing strategies, the addition of a tailored product targeting working adults, and strategic outreach partnerships have all expanded the platform’s reach and ability to engage, with 447,783 unique visitors (447,783/3,000,000, 14.93% of the target population, which includes those who are able to understand and access a website such as mindline.sg independently, including youth, adults, digitally literate older adults; a baseline of 3 million is taken) in the 2 years following launch. Of these visitors, 62.02% (277,727/447,783) of these visitors engage with the site by exploring self-care content, completing exercises, or conversing with the AI chatbot. Furthermore, 10.54% (29,271/277,727) of those users return. The framework around the platform’s development is driven by behavior change goals and unique requirements for mental wellness in Asia.

In this descriptive paper, we outline this framework, which focuses on anonymity, trustworthiness, multiple levels of content engagement, customizability, and tight integration into the local mental health care ecosystem, and we present data and analyses accumulated throughout implementation, culminating in a set of recommendations to guide the development of similar platforms in the region and around the world.

### The mindline.sg Platform

**Figure 1:**
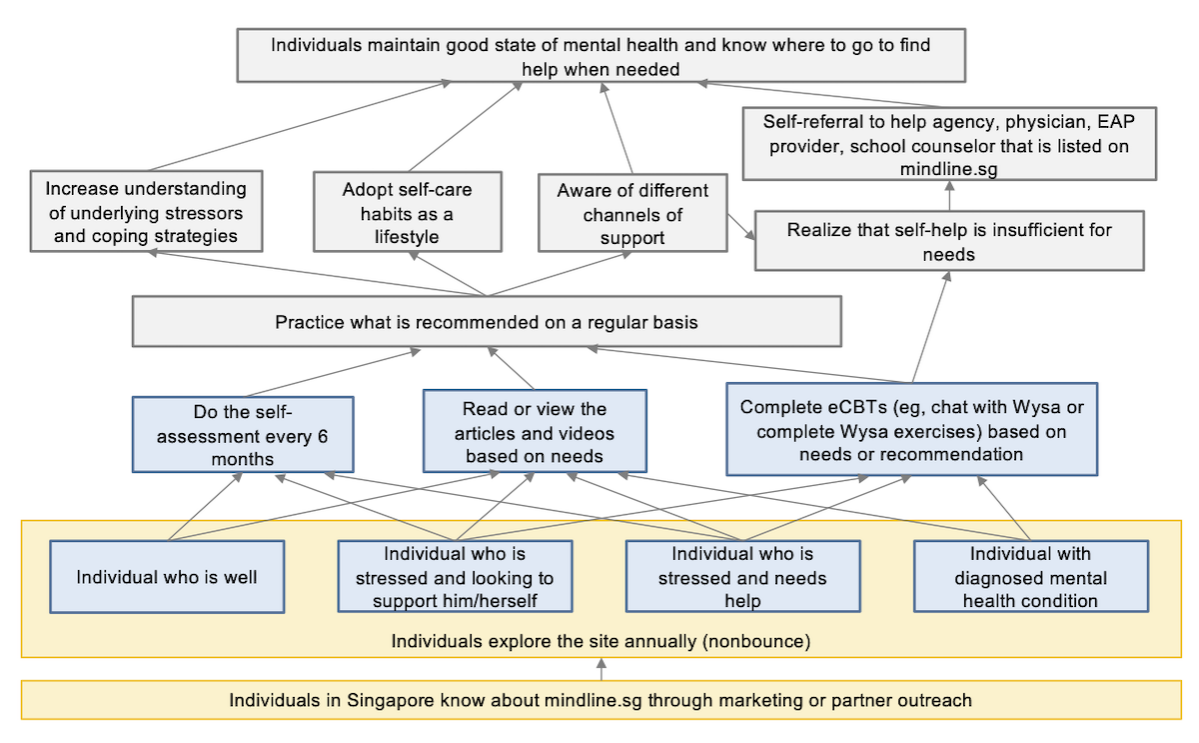
Depiction of the theory of change and goals of the mindline.sg initiative. eCBT: electronic cognitive behavioral therapy; EAP: employee assistance program.

The goals of the platform and the theory of change underlying it can be summarized as described herein. The designers of the platform believe that providing individuals with localized and trusted digital mental health resources that have low barriers to use will lead them to adopt healthy behaviors that maintain their mental wellness. As such, mindline.sg is a stress management and coping website that can be accessed and used anonymously from anywhere for free (the Wysa chatbot component, however, is only licensed for free use within Singapore). The site first exposes individuals to mental health education (when they view content and read articles) and subsequently aims to train them in mental wellness self-management skills (through practicing therapeutic and self-care exercises). The platform recognizes that a wide variety of social, economic, and personal lifestyle factors can cause stress or emotional distress, and therefore, feeling down, anxious, or depressed is something that anyone in the society can encounter at any point in their lives. Therefore, the site includes resources for financial stress, work support, caregiver support, keeping fit and healthy, etc. The goal of the platform is to empower all residents in Singapore to take charge of their mental health and to be able to offer support to those around them; this is accomplished when individuals develop self-care habits and coping skills as a lifestyle and possess the know-how to self-refer to professional services when needed. This theory is summarized in Figure 1. Broader goals to which the initiative may contribute include destigmatizing mental health and increasing mental health literacy. At the time of creating mindline.sg, there were no comparable services available in Singapore or Asia and few global resources offered similar depth and breadth.

## Methods

### A Framework for the Development of a Digital Mental Health Platform

#### A New Framework

We describe a framework for the development of mindline.sg that is centered around anonymity, building trust, progressive engagement levels, and customizability to different population segments. The framework was developed referencing other recognized digital mental health frameworks, such as the American Psychiatric Association’s App Evaluation Framework [19]. Most if not all such frameworks, however, are designed to develop and evaluate mobile apps. As we will discuss, the mindline.sg platform was implemented as a web app to satisfy requirements to reduce barriers to use and increase perceived anonymity. Our framework nonetheless satisfies or fits within the components of existing frameworks for digital mental health apps.

#### Trust and Anonymity

An advisory and editorial board (AEB) consisting of mental health and health care leaders and experts from among the health care ecosystem in Singapore was formed to drive decision-making around platform development and to bring in delivery partnerships and value-adding stakeholders. The AEB also included a clinical review panel consisting of qualified mental health clinicians. Together, the AEB and clinical review panel provided detailed advice; oversight; support; and validation of the clinical effectiveness and safety of the content, referral and escalation methodology, etc, providing assurances to users that the quality of resources and methodologies used are trustworthy.

**Figure 2:**
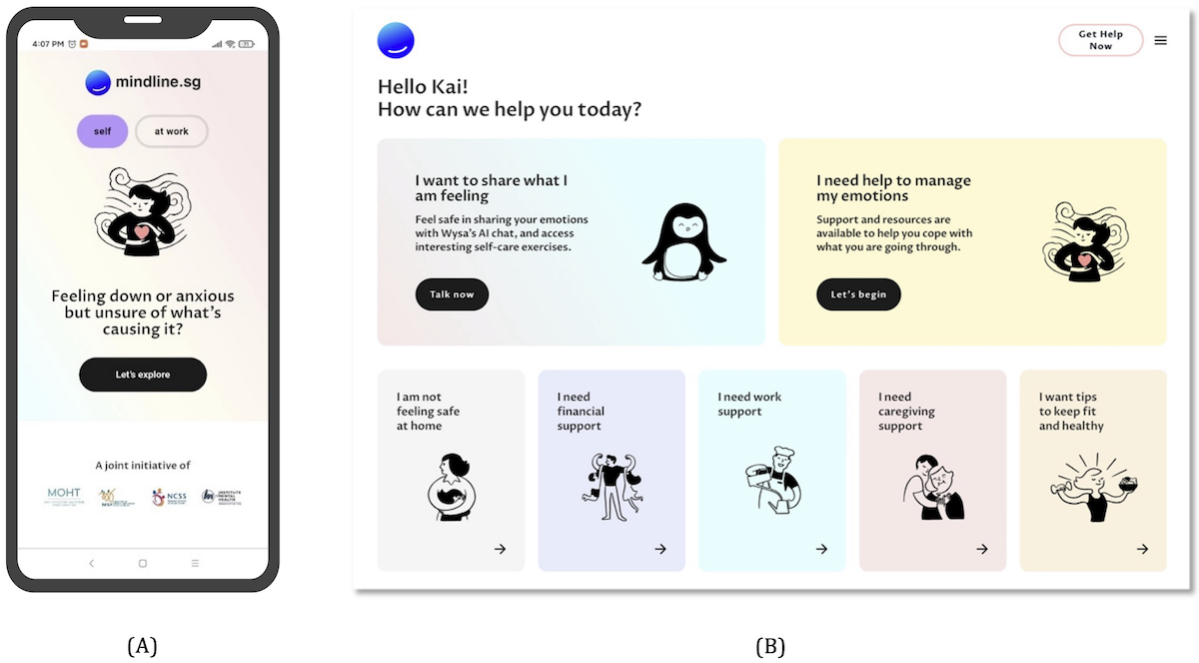
The mindline.sg landing page on (A) a mobile device and (B) the home page on a desktop computer.

Singaporeans have a high level of confidence in the government, with 81.5% of respondents in a World Values Survey study indicating *a great deal of confidence* or *quite a lot of confidence* in the local government [20]. In addition, >70% of survey respondents in Singapore indicated a high confidence in state institutions such as the civil service, as compared to the media or other nongovernment organizations [20]. Therefore, the government agencies behind mindline.sg are highlighted on the website landing page (Figure 2A). While government origins on the landing page will give some users confidence, overt associations with the government could deter others. A generic name and website address mindline.sg was therefore chosen, as opposed to a website address with a .gov.sg suffix.

Stigma remains a challenge for mental health care in Singapore [21-23], perhaps partially explaining the 78.6% treatment gap described in the Introduction section. Moreover, there is a general fear (especially in Asia) of being “labeled” with a mental health condition. Users must therefore be able to access the platform anonymously to minimize barriers to use. A substantial emphasis is therefore placed on anonymity; no personally identifiable data are collected by mindline.sg. Only an anonymous user ID associated with the browser and device that accessed mindline.sg (ie, a cookie ID) is generated and collected by the site, along with an optionally provided nickname (to personally address the user; Figure 2B) or an age range (to personalize the promotion of content). To further emphasize anonymity, the website is accessible through a web app, instead of a mobile app, and requests no registration for use. (We note that this feature may merely improve *perceived* anonymity, as it may be possible to offer an equivalently anonymous experience with mobile apps.)

#### Progressive Engagement for Different Stages of Learning and Readiness for Change: Incorporating a Self-Assessment Tool and Digital Therapeutics Delivered via an AI Chatbot

The platform strategically offers multiple levels of progressively deeper engagement driving behavior change goals. The first step is making users aware of mindline.sg through marketing and outreach efforts or visiting the site. Raising awareness of mindline.sg is a necessary outcome for the initiative. Reach is achieved through search engine optimization, marketing (through both traditional and digital means such as advertisements on social media), and building strategic partnerships (eg, with educational institutes and organizations supporting a profession) to leverage influence with targeted audiences (such as youth and working adults).

The second step is for users to view and read articles, increasing their mental health literacy and awareness of local resources, including channels of support. Curated and trustworthy resources are provided on the site.

**Figure 3:**
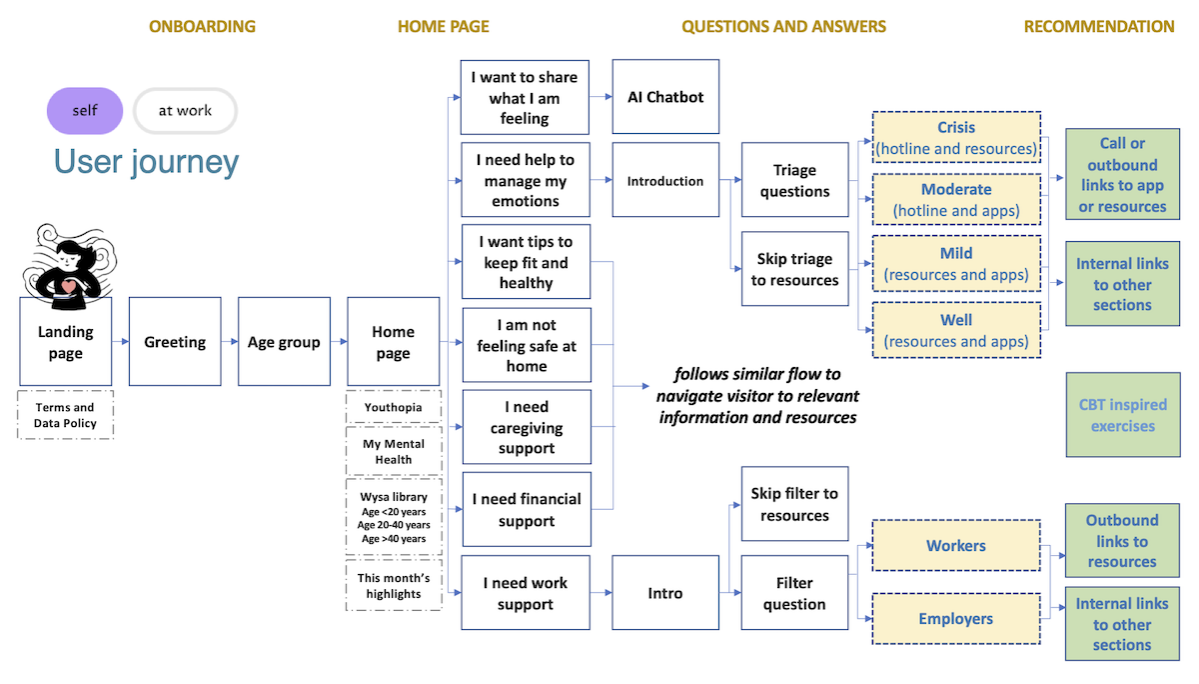
User journey on mindline.sg. The self-assessment wellness protocol determines the resources recommended to the user, including exercises with the artificial intelligence chatbot (for well and mild protocols) and direction to support services (for moderate and severe protocols). AI: artificial intelligence.

**Figure 4:**
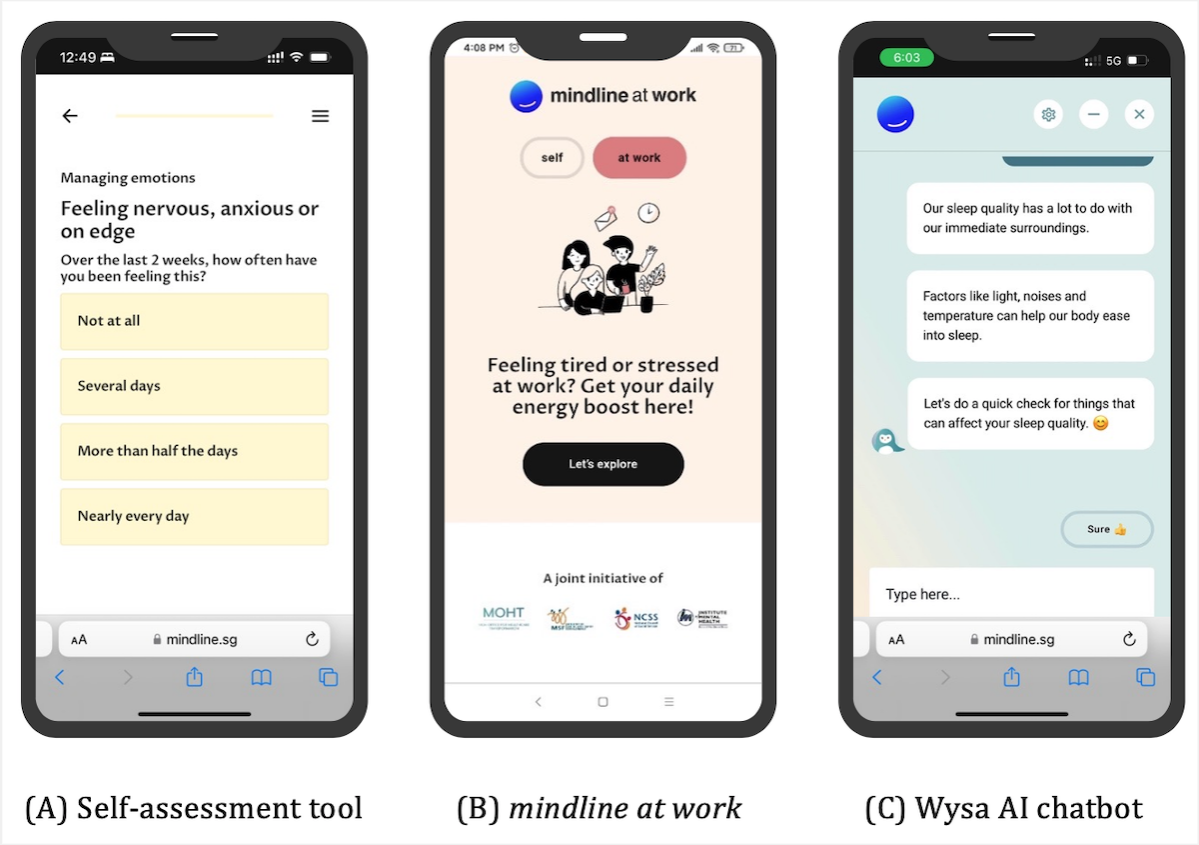
Interventions designed to deepen engagement. (A) A view of the self-assessment tool. (B) The landing page for the mindline at work product customized for working adults. (C) An interactive dialogue-based exercise inspired by cognitive behavioral therapy with the Wysa artificial intelligence chatbot.

The final step is to interactively practice a self-care skill offered on the site. One such skill is self-assessment, for which the site offers a self-assessment tool*,* developed to measure one’s own mental health state easily and accurately so that it may be regularly monitored and acted upon when needed. The tool is composed of the Generalized Anxiety Disorder-7 (GAD-7), which assess anxiety symptoms, and the Patient Health Questionnaire (PHQ; PHQ-9), which assesses depression symptoms and self-harm ideation; these tools are regarded to be well-clinically validated. To be as brief as possible, the tool starts with the ultrabrief instrument PHQ-4 (consisting of the first 2 items of GAD-7 and PHQ-9 each) and only continues onto the rest of the instruments if the user’s responses indicate more than minimal to mild symptoms, according to the instruments’ defined thresholds. The tool ultimately triages the user into 1 of 4 wellness protocols: well, mild, moderate, and in crisis*,* based on symptom severity, which provides the user with an actionable insight and allows the platform to personalize resource recommendation. The self-assessment tool is described in detail in Multimedia Appendix 1. In Figure 3, we show a screenshot of a question in the self-assessment tool. In Figure 4, we show an example user journey on mindline.sg involving the recommendation of resources according to assigned wellness protocol by the self-assessment tool.

Another important source of training offered through the site are digital therapeutic exercises delivered by an AI chatbot from Wysa [24]. This emotionally intelligent chatbot is embedded into the platform and deploys a suite of dialogue-based interactive exercises inspired by cognitive behavioral therapy. These exercises teach skills such as mindfulness, managing stressors, meditation, reframing thoughts, and sleep techniques. Moreover, the chatbot can engage in free-form conversations, providing a pocket therapist for the user to share their emotions in a safe and anonymous manner, anytime and anywhere. The efficacy, safety, and impact of the Wysa chatbot have been evaluated in multiple studies [25]. A customized version of the Wysa web app was used, though could be comparable to Android app versions 0.7.2.8 (September 8, 2020) through 3.0.4 (May 30, 2022), available throughout the duration under analysis. A screenshot of a dialogue session with the Wysa AI chatbot is shown in Figure 4.

#### Directing Users With Moderate Symptom Severity or Who Are in a Crisis to Professional and Emergency Care

The designers of the platform are acutely aware that digitally delivered services have limits and appropriate referral to crisis services must be offered when indicated. Indeed, a key component of mindline.sg was the identification, either through the self-assessment tool or the chatbot, of users who are at risk or in a crisis and directing them to human-based hotlines and emergency services. We take care to clearly publicize these limitations in the following ways: first, at the footer of all mindline.sg platform pages, users are advised to contact emergency services if they are at risk of immediate harm. Second, when the navigation (aka hamburger) menu is opened from any page, a Get Help Now link is immediately visible, which directs individuals to emergency services (including hotlines) and provides information on how to help others in crisis. Third, if a user is self-assessed into the moderate protocol, they are directed to counseling centers and hotlines, with options to launch calls directly from the site. Fourth, if a user is self-assessed into the in-crisis protocol, they are directed to emergency hotlines and a suicide prevention help page. Fifth, if the AI chatbot detects a user in an emergency (such as one expressing self-harm ideation), they are immediately directed to local emergency services.

#### Ensuring Customizability of the Platform: Population Segmentation and mindline at work

mindline.sg was created during the COVID-19 pandemic to address the acute increase in stress and anxiety levels, which were particularly borne by working adults because of job insecurities, new working arrangements, and the lack of usual supporting services (eg, childcare or older adults care). A version of the platform tailored to this subpopulation was therefore added in July 2021 (approximately 1 year following launch), branded as mindline at work. Key differentiating features of the product include mood check-ins (which is an ecological momentary assessment) and user journeys developed around common workplace stressors for both employees (eg, on navigating a toxic work environment) and employers (eg, on nurturing positive workplace cultures). The landing page of the mindline at work product is shown in Figure 4.

This completes the description of the components of the development framework. In Table 1, we provide a summary of the principle mental wellness interventions deployed on the site, the mental wellness goals they intend to serve, and the evidence supporting their use.

**Table 1:** Summary of specific evidence-based interventions deployed on the site along with details on the goals they attempt to serve.

Mental wellness intervention	Goal	Evidence
Curated local mental health resources	Increase mental health literacy and knowledge of stressors and coping strategies	Content from authoritative (evidence-backed) sources curated with help from the clinical review panel and advisory and editorial board
Self-assessment tool (comprising the PHQ-9^a^ and GAD-7^b^ instruments)	Enable users to self-monitor and take action when needed	PHQ-9 and GAD-7 among the most well-validated mental health screening tools
Digital therapeutic exercises delivered through the Wysa chatbot	Provide evidence-based tools to practice self-help skills	Smartphones and internet-based interventions are accessible and effective [9-13] Wysa chatbot is well-validated [25]
Direction to professional services	Ensure users know where to seek help when needed	Site not designed to serve the needs of users with moderate to severe mental health symptoms Digital apps are more effective when used in conjunction with a clinician [26]

^a^PHQ-9: Patient Health Questionnaire-9.

^b^GAD-7: Generalized Anxiety Disorder-7.

### Analytical Methods

While the anonymity features of the site facilitated users’ trust in the platform, they severely limit evaluation efforts to what can be determined from event logs associated with anonymous user IDs. In particular, all usage is captured in a database that records every event, that is, interaction with the site, by the user. These data are further supplemented by Urchin Tracking Module (aka UTM) tags (which are special codes segmenting site traffic) that record the origin of an incoming link (eg, from a targeted advertisement or QR code) and by Google Analytics, which collects inbound activity from other sites. The activity from crawlers (a common method of operating search engines or searching for web resources) is not included in our data.

Unique anonymous user IDs are interpreted as *unique users*, which has the limitation that a single user could be accessing the site from multiple devices or a new anonymous user ID could be assigned when a user clears their browser cache or switches to a different browser. A *bounced* *user* visits the site and performs at most 1 page load. Because the platform serves as content directory rather than repository (users are merely directed to external content, including when engaging with the chatbot), nonbounced users are the most accurate metric available for engagement measurement. An *engaged* *user* is defined as a nonbounced user that spends ≥40 seconds on the site, which indicates a higher level of engagement most likely involving an exploration of multiple resources. A threshold of 40 seconds was chosen during initial site design as an estimate by its designers on how long a user interested in ≥1 resources would likely spend navigating the site or completing an exercise. Note that this is likely to be an underestimate of engagement again because mindline.sg does not host its own content; users could therefore be redirected from the site but engage for far longer than 40 seconds on the linked content. Recent research also estimates the average attention time spent on a digital device screen to be 47 seconds, which is a comparable number [27]. A *return user* is identified by a returning anonymous user ID on another day after the first visit, which again could be inaccurate if users return from a different device or clear their web browser cache. A *session* is defined as contiguous segments of clicks containing pauses (periods of no web activities) of <30 minutes.

We only count users who complete the self-assessment tool and do not exit the assessment early. We can also accurately count users who use the AI chatbot by only counting those who successfully handover to the Wysa platform and engage in a chat.

### Statistical Analyses

We compute conversion rates of users that are nonbounced, that are engaged, and that return and we test whether the distribution of those who engage and return differ significantly between years and product using the Fisher exact test.

### Ethical Considerations

Ethics review was not required because this research involved analysis of data from an anonymous, nonclinical, and global population (though most users are assumed to have been in Singapore). All users of the mindline.sg platform accept the terms of use and data protection policy (copies of which are provided in Multimedia Appendix 2), which states that statistical data on use is collected and may be used for “research and service enhancement.” These policies highlight that these statistical data are anonymous, that the site stores no personally identifiable information, and that the data are transmitted and stored according to industry standards. No individuals were compensated.

## Results

### Site Use and Growth

**Figure 5:**
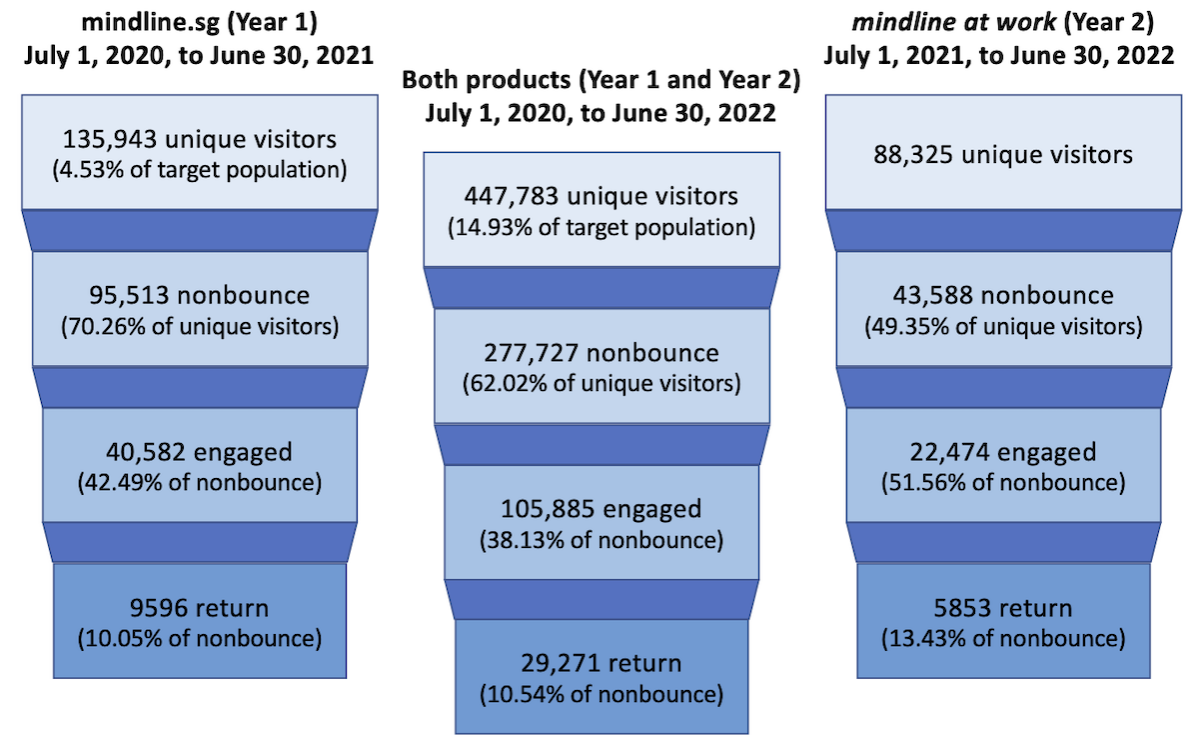
Engagement funnels for mindline.sg and mindline at work during the first year of each product and across the platforms in the 2 years following launch.

The mindline.sg website was launched in June 2020, and mindline at work was launched a year later in June 2021. Visitor and engagement metrics across the platform and for the individual mindline.sg and mindline at work products during these years are summarized as user funnels, depicted in Figure 5.

The following year-on-year growth metrics describe the growth of the platform use from the period July 1, 2020, to June 30, 2021, to the period July 1, 2021, to June 30, 2022, across both sites:

- There was a 126% growth year-on-year in total unique visitors (from 135,943 to 447,783).
- There was a 39.7% growth year-on-year in nonbounced users (from 95,513 to 277,727).
- There was a 59% growth year-on-year in engaged users (from 40,582 to 105,885).
- There was a 110% growth year-on-year in return users (from 9596 to 29,271).

The platform’s 126% year-on-year growth rate is high. For reference, a marketing firm reported that nonprofit and health care websites studied achieved year-on-year growth rates of 70% and 113%, respectively [28].

The median duration spent on either the mindline.sg or mindline at work sites by nonbounced users during the first 2 years was approximately 137 seconds. As noted in the Analytical Methods section, this is not a good measure of engagement, as the time spent off the site on externally hosted resources is not known. This duration is merely a measure of time spent navigating around different resources on the site.

### Development of the Analytics Dashboard

**Figure 6:**
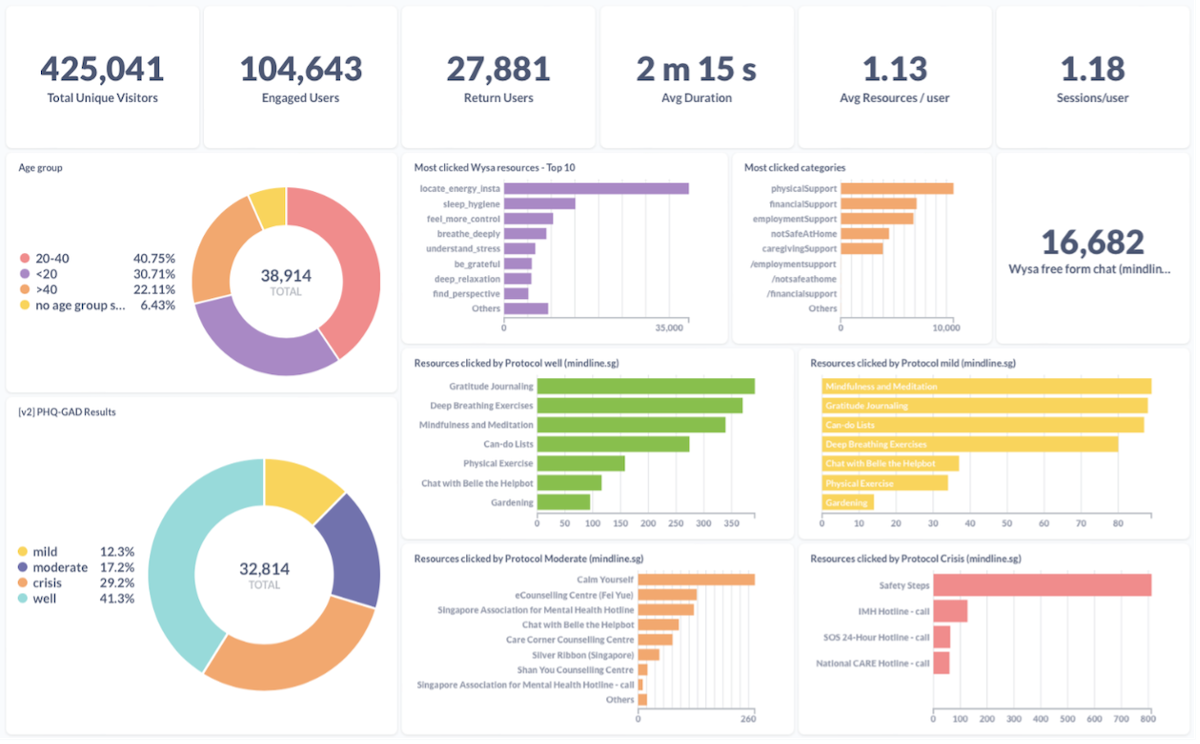
Customizable analytics dashboards used to continually drive process evaluation, digital marketing strategy, and site redesign.

During implementation, an analytics dashboard for click-data analytics was implemented using Metabase [29], a commercial and open-source platform for data visualization and business intelligence. One such dashboard is shown in Figure 6. These dashboards were essential in driving digital marketing strategy and providing customized analytics to strategic partners. The underlying datastore was Titanium Database [30], an open-source hybrid transactional and analytical processing database, running on a 10-node Kubernetes cluster on the cloud.

### Digital Marketing

**Figure 7:**
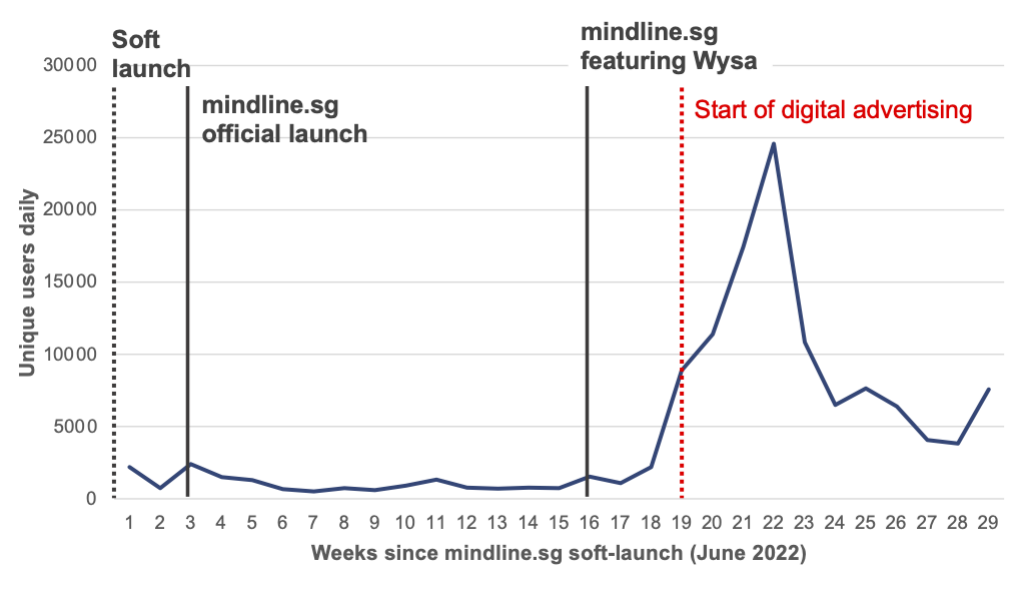
Number of weekly users to mindline.sg before and after digital marketing.

Data analytics showed a strong relationship between publicity efforts (ie, newspaper advertisement, radio advertisement, or Facebook advertisement) and visitors to mindline.sg, as seen in Figure 7. Digital marketing, that is, advertising to potential users through digital channels ranging from search platforms to social media platforms, effectively reaches out to population groups who are more likely to use and explore a site such as mindline.sg and to use products such as the AI chatbot. The Analytics Dashboard was analyzed daily to dynamically drive the placement of advertisement campaigns.

Digital marketing campaigns focused on the placement of advertisements onto social media platforms, including Facebook, Instagram, Google, Reddit, and YouTube. Advertisements usually took the form of static graphics, though occasional video advertisements were used. Advertisements were placed using a combination of placements through advertising platforms, including Taboola and Outbrain, and custom advertisement placements. These advertisements often targeted a common stressor, for example, with invitations to users to “improve sleep” and “locate energy,” and often linked directly into a resource on the site such as a Wysa therapeutic exercise. Micro influencers with strategically targeted audiences were also engaged to promote the site and advertisement content.

### Use of the AI Chatbot and Therapeutic Exercises

The Wysa AI chatbot was added to the site in October 2020. Through June 30, 2022, a total of 67,626 unique users (which is 25.54% of the 264,758 nonbounced visitors to the platform over this same period) engaged with the chatbot through a cognitive behavioral therapy–inspired exercise or a free-form chat. As a measure of meaningful engagement, around 75.6% (51,125/67,626) of these users exchanged >5 messages with the chatbot. A completion rate of 74.5% (50,381/67,626) was achieved for all therapeutic exercises initiated.

By comparison, 11.69% (32,469/277,727) and 9.52% (26,434/277,727) of the nonbounced visitors completed a self-assessment exercise or viewed some other resource (such as an article or video) through the first 2 years on the site, respectively. The therapeutic exercises delivered by the AI chatbot are therefore the most popular feature of the site. The most popular exercises were energy maintenance, feeling in control, understanding stress, sleep hygiene, breathing, and relaxation.

### Self-Assessment Use and Behaviors by Wellness Protocols

Taking the self-assessment questionnaire has consistently proven to be one of the most popular activities on the site. During the 2-year period from July 1, 2020, through June 30, 2022, a total of 41,366 self-assessments have been completed from among 32,469 unique individuals (11.69% of the 277,727 nonbounced visitors over this period). This makes it the second most popular feature on the site after the suite of exercises offered by the chatbot.

Among the set of final self-assessments completed across the users, 44.82% (13,403/32,469) were assigned to the well protocol, 12.28% (3986/32,469) to the mild protocol, 17.26% (5604/32,469) to the moderate protocol, and 29.18% (9476/32,469) to the crisis protocol.

Of the users assigned to the well and mild protocols, 13.01% (1744/13,403) and 10.76% (429/3,986), respectively, explored the resources recommended to them. The most popular of these were gratitude journaling, mindfulness, meditation, and deep breathing exercises. Users assigned to the moderate protocol were slightly more engaged, with 14.72% (825/5604) exploring the resources recommended to them. These included 52.5% (433/825) of those who further engaged, navigating to professional support services, including counseling centers and a mental health hotline. Finally, 11.21% (1062/9476) of the users assigned to the crisis protocol engaged with a recommended resource. Moreover, 23.54% (250/1062) of these individuals elected to launch a call immediately to one of the linked emergency care hotlines. However, most of the individuals (812/1062, 76.46%) in this protocol that further engaged elected to instead read an emergency support page for those experiencing suicidal ideation.

### Use Behaviors in mindline at work

Feedback from surveys, focus group discussions, and the AEB drove cocreation of the mindline at work product and its curated resources. The visitor and engagement metrics can be seen in Figure 5.

Conversion rates (ie, rates of successful engagement) used to compare engagement between the 2 sites are shown in Table 2. Digital marketing efforts were substantially increased during year 2, resulting in a large influx of new visitors and a subsequently lower nonbounced user rate. As can be seen in Table 2, the engaged and return user rates of 51.56% (22,474/43,588) and 13.43% (5853/43,588) of the nonbounced users, respectively, were notably higher on mindline at work than they were on mindline.sg during its comparable first year period, which were 42.49% (40,582/95,513) and 10.05% (9596/95,513), respectively. By comparison, the mindline.sg site saw 30.9% (42,829/138,626) and 9.97% (13,822/138,626) engaged and return user rates in the second year, respectively. The Fisher exact test (with test statistic *H* and *P* value) confirmed that both the increases in engaged and return users on the mindline.sg site from year 1 to year 2 were significant (engaged: *H*=0.73; *P*<.001 and return: *H*=1.14; *P*<.001). This could be explained by the increased strategic outreach initiatives to target user groups in the second year, which brought users that were more likely to engage with the platform. Similarly, the Fisher exact test confirmed that both engaged and return user rates on the mindline at work platform were significantly higher than those on the mindline.sg platform during that same year (engaged: *H*=1.96; *P*<.001 and return: *H*=1.21; *P*<.001). We conclude that the mindline at work site achieved better engagement among its target population.

**Table 2:** User conversion rates in the first and second years of mindline.sg and in the first year of mindline at worka.

Conversion rates	mindline.sg year 1, n (%)	mindline.s*g* year 2, n (%)	mindline at work year 2, n (%)
Nonbounced rate (of all visitors)	95,513 (70.26)	138,626 (62.02)	43,588 (49.35)
Engaged user rate (of nonbounced users)	40,582 (42.49)	42,829 (30.9)	22,474 (51.56)
Return rate (of nonbounced users)	9596 (10.05)	13,822 (9.97)	5853 (13.43)

^a^Year 1 denotes the period July 1, 2020, to June 30, 2021, and year 2 denotes the period July 1, 2021, to June 30, 2022. The nonbounced rates seen in year 2 are lower than those in year 1 due to substantially larger numbers of visitors because of increased digital marketing efforts. We see that the mindline at work site has higher engaged user and return user rates than the mindline.sg site.

## Discussion

### Limitations of This Study

The site only collects anonymous user IDs to maintain the level of anonymity required. A major limitation of this feature, however, is that no demographic or other user data are collected, substantially limiting any ability to understand user profiles and to evaluate the impact of the platform. These limitations have already been detailed when defining important metrics in the Analytical Methods section.

Another limitation is that some users could be adopting exploratory behaviors on the site out of curiosity, for example, artificially triggering different wellness protocols during the self-assessment. While this might result in some statistical noise, we do view exploration of the site and the range of resources available for different situations to be a desirable and healthy behavior. To mitigate any impact of this effect on our data analyses, we have taken appropriate data processing steps; for example, we discard all repeated self-assessments in a single session.

### Trust and Anonymity

To serve the goal of building a site that users trust in the local Singaporean context, both in terms of content reliability and on anonymity associated with use, the site designers curated content and tools that were endorsed by both local experts in the field and the government. While Singaporeans generally have a high level of public trust in the government (as detailed in the Methods section), this may not be so for all communities.

While the promise of anonymity aims to build trust with users, the anonymity requirements restricted use metrics to anonymous user ID–based data. Evaluation and personalization were therefore difficult without detailed tracking of user-level data. Future iterations of the platform should include a framework for anonymous user profiling.

### Marketing and Outreach for Awareness

A key learning from implementation is that digital well-being solutions aimed at population-level intervention require substantial and systematic marketing and outreach efforts to achieve desired reach and economies of scale.

From early process evaluation efforts, it was learned that an extensive and customizable, yet still user-friendly, analytics infrastructure was needed to support digital marketing and outreach partnerships, and the analytics dashboard was therefore implemented. Future iterations of the platform must therefore aim to drive user growth with data-driven digital marketing and ecosystem partnerships, which must be supported by such an appropriate analytics infrastructure.

### Progressive Engagement

The progressive engagement approach on mindline.sg encourages users to initially explore the site and read articles, followed by deeper engagement practicing self-care skills through tailored products, nudges, and personalization. Therapeutic exercises delivered through the AI chatbot are the most used feature on the site, indicating that digital therapeutics that enable users to practice skillsets adds immense value. However, we cannot confidently rule out that the AI chatbot appeared to be the most used feature as people tried it more out of curiosity than actual need and so did not engage meaningfully with it. We note that AI chatbots are generally targeted toward those who are well or have only mild symptoms.

The self-assessment tool was the second most used feature on the platform, with 11.69% (32,469/277,727) of the nonbounced users completing at least 1 self-assessment. This is comparable to the uptake seen on another digital mental health platform, Beyond Blue (run by the Australian government), which saw approximately 6.5% and 17.4% of the nonbounced users complete the GAD-7 instrument and a custom 10-item anxiety and depression checklist on their site in 2017 (we have computed these estimates based on data made available in an evaluation by Siggins Miller Consultants [31] and from a report by Beyond Blue [32]). We have learned that users like to assess themselves; there could be an implicit interest in benchmarking oneself against others. Future iterations could perhaps reflect individual assessments against aggregate results from the assessments to further nudge those who may be in the moderate-to-crisis protocols to seek help. Because this self-assessment tool is derived from existing, validated instruments and is low cost to deploy and scale digitally, it has the potential to be a cost-effective intervention for the platform.

### Customization and Personalization to Increase Engagement

While most users were self-assessed to be well or have only mild symptoms, a substantial proportion were self-assessed to have moderate symptoms or were in crisis, with 29.18% (9476/32,469) falling under the crisis protocol. Although we surmise that some of these users may be triggering the crisis protocols out of curiosity, we have no independent assessment to determine the actual rate, other than numerous press and scholarly reports that have suggested that mental anguish is greater than generally believed. We can conclude that mindline.sg is attractive to people at all levels of symptom severity, and this is supported in part by the fact that those in the moderate protocol were found to be slightly more committed to help seeking in their follow-up actions after self-assessment. The platform therefore has the potential to further drive help-seeking behavior in users with moderate to severe symptoms and should link such users to human-based support within the platform itself (eg, community forums and therapists) as well as outside the platform (eg, telemedicine and clinical consults).

On the basis of our customization experience for a more specific population segment with mindline at work, we found that the tailored product for working adults deepened the level of user engagement. The hypothesis that customization could help create more targeted, meaningful impact to the mental well-being of specific populations could be tested further with other populations that have been identified to have greater mental health needs, especially since the COVID-19 pandemic, such as youth and health care workers.

### Scaling Digital Mental Health Initiatives and Summary of Recommendations

An estimated 12 full-time employment staff were involved in the development and maintenance of the initiative over the first 2 years.

A summary of our design principles, key features, learning points, and key recommendations from the initial implementation of mindline.sg can be found in Table 3.

**Table 3:** Summary of the design principles, key features, learning points, and key recommendations for mindline.sg.

Design principles and key features			Learning points		Key recommendations
**Design 1 (trust): built as a trusted source for high-quality mental health information and resources through cocreation methodologies**					
	Participatory approach with feedback from service providers and intended users Expert input and oversight through an advisory and expert board Government endorsed	Cocreative methodologies, along with endorsement from multiple authoritative entities, build trust and facilitate acceptance of the digital intervention by a diverse group of people, which would be unlikely if purely government endorsed		Maintain participatory approach to keep enhancing the anonymous user experience Maintain credibility of content that is overseen by expert advisors Obtain endorsement by multitude of authoritative figures or organizations with high public trust.	
**Design 2 (anonymity): maintaining anonymity to encourage convenient help seeking**					
	No collection of personally identifiable data Developed as a web app rather than a mobile app	The promise of anonymity contributed to building trust A web app proved to be easy to use Anonymity severely limits evaluation		Develop a framework for anonymous user profiling Remain as a web app to maintain convenience and anonymity	
**Design 3 (progressive engagement): progressive engagement to facilitate users’ different stages of learning and readiness for change**					
	A robust branding and marketing strategy to raise awareness about the existence of mindline.sg	Substantial digital marketing and strategic outreach partnerships are required to achieve desired reach and economies of scale.		Drive user growth with data-driven digital marketing and ecosystem partnerships, which must be supported by an extensive and customizable analytics infrastructure.	
	Tools to improving mental health literacy Links to curated, expert-endorsed articles and videos for self-learning from local and international sources Self-assessment tool with personalized resource recommendations through expert-developed algorithms Links to local resources and helplines AI^a^ chatbot to guide therapeutic exercises	The substantial proportion of resource use attributable to the AI chatbot indicates that therapeutic content on practicing skillsets adds immense value Users like to assess themselves; there could be an implicit interest in benchmarking oneself against others mindline.sg was attractive to people with varying levels of symptom severity, including those with moderate to severe needs.		Existing features should be retained for their usefulness to users with different needs, though some expansion of features should be considered, for example: Reflect individual assessments against aggregate results for contextualization. Provide deeper links to human-based services from within the platform for those who need further support, for example, linkage to therapy, telemedicine, and community forums	
**Design 4 (customizability): ensuring customizability of the platform for a targeted approach to improving mental health outcomes**					
	Adapting features on the platform that can be tailored to different population segments, for example, mindline at work for working adults	The customized product for working adults deepened the level of engagement		Explore customizations for a variety of population segments with mental health needs to deepen impact with a targeted approach, for example, with youth and health care workers	

^a^AI: artificial intelligence.

### Relevance of the Findings

The theory of change for the platform (described in the Introduction section) requires reach and engagement to be achieved so that users are aware of the resources available on mindline.sg and they gain experience practicing self-care skills. This paper provides evidence that such necessary processes have been accomplished. This supports the subsequent study on whether the desired outcomes (that users practice self-care skills as a habit, access resources when in need, and self-refer to professional mental health services if required, among others) are being achieved. We leave this work to ongoing evaluation studies.

### Conclusions

The mindline.sg digital mental wellness initiative aims to empower all residents in Singapore to maintain their own state of mental wellness, to be able to support others around them, and to self-refer to professional mental health services when needed. This study provides evidence that the initiative has successfully achieved substantial reach and engagement in the 2 years since its launch. Achieving these necessary outcomes required substantial and sustained investment into digital marketing as well as multiple strategic outreach partnerships. The development of an extensive and customizable data analytics infrastructure was required to support these efforts. A self-assessment tool designed for self-monitoring and content personalization, as well as a suite of digital therapeutic exercises delivered by the Wysa AI chatbot, successfully engaged users and were the 2 most popular features of the site. The creation of a customized version of the site for working adults, mindline at work, also achieved higher engaged in its first year compared to the generic mindline.sg site. We have provided recommendations for revision, and we believe this report may help to guide the development of similar platforms around the world for communities with similar needs. With this successful process evaluation, the initiative should now proceed to conduct impact evaluation studies around its desired outcomes.

## Appendix

Multimedia Appendix 1 Description of the self-assessment and triaging tool.

Multimedia Appendix 2 Terms of use and data protection policy for the mindline.sg platform.

## Abbreviations

AEB: advisory and editorial board
CRP: clinical review panel
GAD-7: Generalized Anxiety Disorder-7
PHQ: Patient Health Questionnaire

## Data Availability

The data sets generated during and analyzed during this study are not publicly available due to the mindline.sg data protection policy but are available from the corresponding author on reasonable request.
