# Supplementary material for: Mental Wellness Self-Care in Singapore With mindline.sg: A Tutorial on the Development of a Digital Mental Health Platform for Behavior Change
Source: J Med Internet Res. 2024 Jun 4;26:e44443. doi: 10.2196/44443 (PMC11185903; doi:10.2196/44443)
Supplement: Multimedia Appendix 1 [file jmir_v26i1e44443_app1.docx]

## Multimedia Appendix 1

## Description of the self-assessment and triaging tool

The development of the tool satisfied the following requirements.

- The tool must be brief (to encourage regular use), so it is designed as an adaptive questionnaire, expanding in scope only when needed.
- The tool must screen for a range of symptoms and severity (to serve the wide range of users coming to mindline.sg) -- so the questionnaire comprises items from *GAD-2* and *GAD-7*, components of the *Generalised Anxiety Disorder Assessment*, which screens mild to severe anxiety symptoms, and *PHQ-2* and *PHQ-9*, components of the *Patient Health Questionnaire*, which screens minimal to severe depression symptoms. These instruments are self-administered and do not require professional supervision.
- The tool must be evidence-based and reliable, so the algorithmic assembly of the GAD and PHQ components (which are themselves widely validated) was developed in close consultation with the AEB.
- Finally, the results of the tool must be easy to understand and provide actionable insights, so the tool assigns users to one of four *wellness protocols*: *well*, *mild*, *moderate*, and *crisis*, which also serves to triage users so that they may be directed to appropriate resources.

The PHQ-9 questionnaire consists of nine items measuring the experience of depression criteria, each assigning a score from zero (“not at all”) to three (“nearly every day”). The total score ranges from zero to 27 with recommended cut-off points of 5, 10, 15, and 20 for mild, moderate, moderately severe, and severe depression, respectively. GAD-7 is an analogous questionnaire that consists of seven items measuring anxiety, with a total score ranging from zero to 21 and recommended cut-offs of 5, 10, and 15 for mild, moderate, and severe anxiety, respectively.

The self-assessment tool begins with the first two items of both GAD-7 and PHQ-9, which are referred to as *GAD-2* and *PHQ-2*, respectively (and which together are referred to as *PHQ-4*, itself a validated instrument). The rest of the adaptive questionnaire is then determined by the following algorithm.

1. **Minimal anxiety and depression:** If the user's GAD-2 and PHQ-2 scores are both less than three, then they are assigned to the *well* protocol.
2. **Minimal depression and potential anxiety:** If the user’s GAD-2 score is three or greater, then their anxiety symptoms should be further investigated, and if the user has no or minimal depression symptoms (PHQ-2 score less than three), then they are presented with the remaining five items of the GAD-7 questionnaire.
   1. **Minimal anxiety:** If the user’s total GAD-7 score is less than five, then they are assigned to the *well* protocol.
   2. **Mild anxiety:** If the user’s total GAD-7 score is between five and nine, inclusive, then they are assigned to the *mild* protocol.
   3. **Moderate to severe anxiety:** If the user’s total GAD-7 score is 10 or more, then they are assigned to the *moderate* protocol.
3. **Potential depression:** If the user’s PHQ-2 score is three or greater, then their depression symptoms are further investigated regardless of their GAD-2 score, and they are presented with the remaining seven items of the PHQ-9 questionnaire.
   1. **Minimal to mild depression:** If the user’s total PHQ-9 score is less than 10, then they are assigned to the *mild* protocol.
   2. **Moderate to moderately severe depression:** If the user’s total PHQ-9 score is between 10 and 19, inclusive, then they are assigned to the *moderate* protocol.
   3. **Severe depression and/or self-harm/suicidal ideation:** If the user’s total PHQ-9 score is 20 or greater or if they answer positively to the item investigating suicidal ideation or self-harm, then they are assigned to the *crisis* protocol.

The following is a summary of the interpretation of the four protocols, along with a description of the content to which their assigned users are subsequently directed.

- **Well:** *The user has minimal depression and anxiety symptoms*. Users in both this and the mild protocols are presented with resources and digital therapy exercises that help with the maintenance of their mental well-being and are otherwise invited to explore the site.
- **Mild:** *The user has mild anxiety symptoms and/or minimal to mild depression symptoms*.
- **Moderate:** *The user has moderate to severe anxiety symptoms and/or moderate to moderately severe depression symptoms*. Information and contact details for a variety of professional service providers (e.g., counselling centres) are presented, followed by digital therapy tools.
- **Crisis:** *The user has severe depression and/or has expressed self-harm or suicidal ideation*. The user is immediately directed to local emergency resources, including a 24-hour hotline.

The self-assessment tool is visually summarised in Figure 1.

| 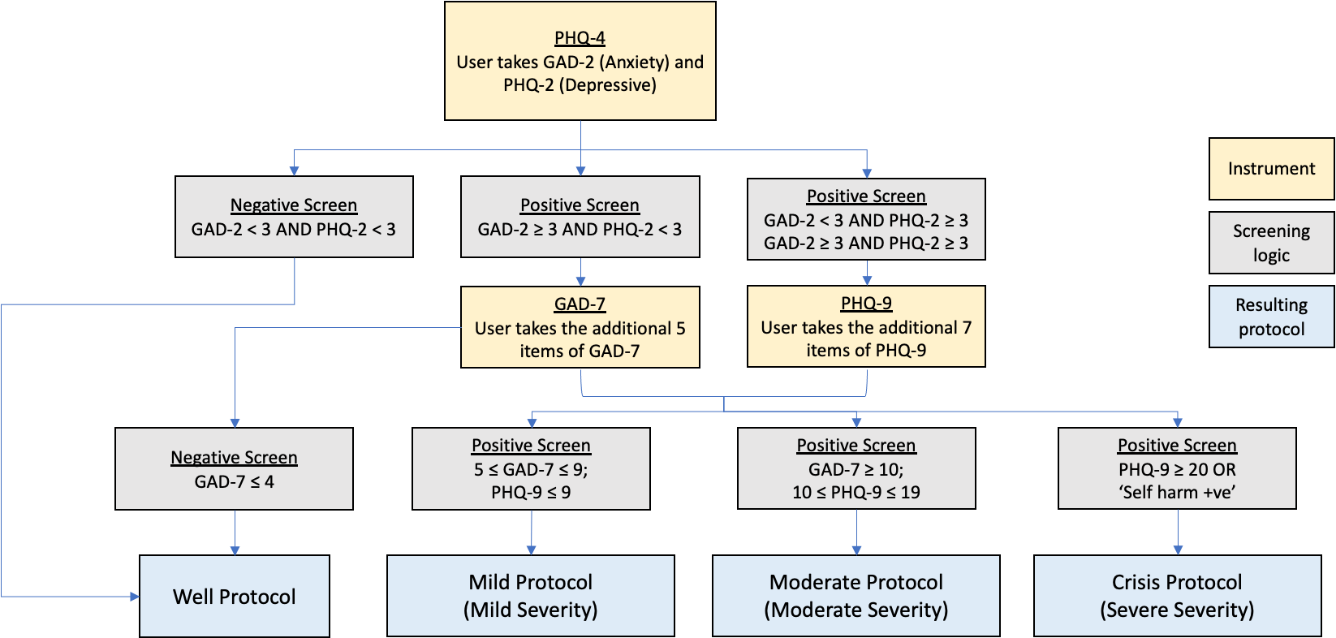 |
| --- |
| Figure 1. Depiction of the self-assessment and triage protocol algorithm. |
