# Supplementary material for: Mental Wellness Self-Care in Singapore With mindline.sg: A Tutorial on the Development of a Digital Mental Health Platform for Behavior Change
Source: J Med Internet Res. 2024 Jun 4;26:e44443. doi: 10.2196/44443 (PMC11185903; doi:10.2196/44443)
Supplement: Multimedia Appendix 2 [file jmir_v26i1e44443_app2.docx]

## Multimedia Appendix 2

## Terms of Use and Data Protection Policy for the *mindline.sg* platform

### **TERMS OF USE**

IMPORTANT NOTICE:

PLEASE READ THESE TERMS OF USE ("TERMS OF USE") CAREFULLY BEFORE USING THE MINDLINE.SG WEB APP WEBSITE, APP, SYSTEM, TOOL AND/OR ITS RELATED MATERIALS AND CONTENTS THEREOF (HEREINAFTER REFERRED TO AS THE “MINDLINE.SG WEB APP”).

BY ACCESSING OR USING THE MINDLINE.SG WEB APP, YOU UNCONDITIONALLY ACCEPT AND AGREE TO BE BOUND BY THESE TERMS OF USE. IF YOU DO NOT AGREE TO THESE TERMS OF USE, DO NOT USE THE MINDLINE.SG WEB APP.

IN THE EVENT OF A MEDICAL EMERGENCY PLEASE CALL EMERGENCY SERVICES. The mindline.sg web app is not designed or intended for use in the diagnosis or treatment of disease or other conditions, and the recommendations or information provided by the mindline.sg web app does not constitute medical advice. Please consult your healthcare provider prior to making any decisions related to your health. You will be solely responsible for all decisions and actions taken or not taken involving medical diagnosis, treatment and care resulting from or in any way related to the use of the mindline.sg web app.

The mindline.sg web app is only intended for use in Singapore.

1. General

1.1 The mindline.sg web app is made available, not sold, to you by the MOH OFFICE FOR HEALTHCARE TRANSFORMATION PTE LTD (MOHT) for use only under these Terms of Use. MOHT retains ownership of the mindline.sg web app itself and reserve all rights not expressly granted to you.

1.2 MOHT reserves the right to revise this Terms of Use at any time it deems fit by updating this page. You should visit this page from time to time and review the current Terms of Use. MOHT may modify any information that forms part of the mindline.sg web app any time, without the need of notice to you, and without any liability on the part of MOHT. Your use of the mindline.sg web app after such changes have been made available will constitute your unconditional agreement to the modified Terms of Use and all of the changes.

1.3 MOHT, at its discretion, may make available future updates to the mindline.sg web app itself. The mindline.sg web app updates, if any, may not necessarily include all existing mindline.sg web app features. The terms and conditions of these Terms of Use will govern any mindline.sg web app updates, unless such update is accompanied by a separate agreement, in which case you agree that the terms of that agreement will govern such update.  MOHT further reserves the right, at MOHT’s sole discretion, to remove the mindline.sg web app from access or absorb it into other online capabilities.

2. Proprietary Rights

2.1 The mindline.sg web app and its contents and materials made available on or through the mindline.sg web app, are protected by copyright, trademark and other forms of intellectual property rights. All rights in the mindline.sg web app are owned by, licensed to, managed and/or controlled by MOHT.

2.2 Other terms of use, as may be indicated to you may apply upon using this mindline.sg web app or in the event you are redirected to other contents including websites, apps and/or tools. Such content may be protected by rights, copyright or other intellectual property laws and treaties, and may be subject to terms of use of the third party providing such content. The aforesaid third party sites, apps, tools, contents and materials, may not be owned or controlled by MOHT and MOHT shall have no responsibility for their correctness or availability. In addition, MOHT shall not be liable to you or any third party for any damage or loss whatsoever arising from your access to or use of such third party sites, tools and materials.

3. Restrictions on use

3.1 MOHT grants you a revocable, non-exclusive right to access and use the mindline.sg web app subject to the following conditions:

a. The mindline.sg web app is not a medical device and should not be used as a substitute for qualified medical advice. It is not designed or intended for use in the diagnosis of disease or other conditions, or in the cure, mitigation, treatment, or prevention of any condition or disease. Please consult your healthcare provider prior to making any decisions related to your health. You will be solely responsible for all decisions and actions taken or not taken involving medical diagnosis, treatment and care resulting from or in any way related to the use of the mindline.sg web app.

b. You may access and use the mindline.sg web app in the manner permitted in these Terms of Use only. Except as otherwise provided in this Terms of Use, the mindline.sg web app may not be copied, distributed, reproduced, republished, uploaded, transmitted, or otherwise modified in any way, without the prior written permission of MOHT.

c. You may use the mindline.sg web app solely for your personal, non-commercial use. You shall not rent, lease, lend, sell, trade, distribute or license the mindline.sg web app.

d. You shall not use the mindline.sg web app for the principal purpose of advertising or promoting a particular product or service without the prior written permission of MOHT.

e. You shall not use the mindline.sg web app in a way that suggests any official status or that MOHT or MOHT’s contributors endorses you or your use of the mindline.sg web app without MOHT’s prior written consent.

f. You shall not make any deletions, additions, adjustments, alterations, adaptations or modifications to the mindline.sg web app. You acknowledge and agree that any deletion, addition, adjustment, alteration, adaptation or modification of this the mindline.sg web app is a violation of intellectual property rights and other related rights subsisting therein.

g. You must secure permission from MOHT prior to hyperlinking to, or framing, the mindline.sg web app or any of its contents, or engaging in similar activities. MOHT reserves the right to impose conditions when permitting any hyperlinking to, or framing of the mindline.sg web app or any of its contents.

h. You agree that you will comply with the Personal Data Protection Act 2012 (“PDPA”) and/or any other applicable law relating to data privacy or confidentiality, and will not cause MOHT to be in breach of such laws.

i. You may not, and you agree not to or enable others to, copy, decompile, reverse engineer, disassemble, attempt to derive the source code of, decrypt, modify, or create derivative works of the mindline.sg web app or any part thereof.

j. You agree to use the mindline.sg web app in compliance with all applicable laws.

4. Disclaimers against Warranties, Representations and Liabilities

4.1 The mindline.sg web app is provided on an “as is” and “as available” basis without warranties of any kind. To the fullest extent permitted by law, MOHT does not make any representations or warranties whatsoever and hereby disclaims all express, implied and statutory warranties of any kind to you or any third party, whether arising from usage or custom or trade or by operation of law or otherwise, including but not limited to the following:

a. any representations or warranties as to the accuracy, completeness, reliability, timeliness, currency, quality or fitness for any particular purpose of the mindline.sg web app; and

b. any representations or warranties that the mindline.sg web app shall be error-free or shall be available without interruption or delay, or that any defects shall be rectified or corrected, or that the mindline.sg web app is and will be free of all viruses and other harmful elements.

4.2 MOHT shall not be liable to you or any third party for any damage or loss whatsoever, including but not limited to direct, indirect, punitive, special or consequential damages, loss of income, revenue or profits, lost or damaged data, or damage to your computer, software, modem or other property, arising directly or indirectly from:

a. your access to or use of the mindline.sg web app;

b. any loss of access to or use of the mindline.sg web app, howsoever caused;

c. any inaccuracy or incompleteness in, or errors or omissions arising from the mindline.sg web app;

d. any delay or interruption in the transmission of the mindline.sg web app, whether caused by delay or interruption in transmission over the internet or otherwise; or

e. any decision made or action taken by you or any third party in reliance upon the mindline.sg web app, regardless of whether MOHT has been advised of the possibility of such damage or loss.

4.3. You shall not rely on the mindline.sg web app to claim or assert any form of legitimate expectation against MOHT, whether procedural or substantive in nature, in respect of any action that MOHT may or may not take in the exercise of its discretion.

4.4. CAUTION: YOU FURTHER ACKNOWLEDGE THAT THE MINDLINE.SG WEB APP, RECOMMENDATIONS, AND RELATED SERVICES SHOULD NOT BE RELIED UPON AS A SUBSTITUTE FOR QUALIFIED MEDICAL ADVICE. AND THE MINDLIND.SG WEB APP, RECOMMENDATIONS, AND RELATED SERVICES ARE NOT INTENDED OR SUITABLE FOR USE IN SITUATIONS OR ENVIRONMENTS WHERE THE FAILURE OR TIME DELAYS OF, OR ERRORS OR INACCURACIES IN THE RECOMMENDATIONS, CONTENT, DATA OR INFORMATION PROVIDED BY, THE MINDLINE.SG WEB APP COULD LEAD TO DEATH, PERSONAL INJURY, OR SEVERE PHYSICAL HARM OR ANY FORM OF HARM OR DAMAGE.

5. Indemnity

5.1 You agree to hold harmless and to fully indemnify MOHT and/or MOHT contributors to the development and operation of the mindline.sg web app (such MOHT contributors as determined by MOHT), from any and all claims, demands, losses, liabilities, costs, and expenses (including but not limited to legal costs) against MOHT and/or MOHT contributors arising directly or indirectly from your use of this website, your breach of this Terms of Use, or any infringement claim by any third party resulting in damage and/or loss to MOHT and/or MOHT contributors, as the case may be.

6. Availability, Right of Access, Changes and Contents

6.1 MOHT may at any time terminate or suspend the availability, access, operation of the mindline.sg web app or all or any of its contents for any period of time without any prior notice, whether for the purposes updating and upgrading or system maintenance or otherwise.

6.2 MOHT reserves all rights to deny or restrict access to the mindline.sg web app by any person, or to block access from a particular internet address, at any time without prior notice and without ascribing any reason whatsoever.

6.3 The mindline.sg web app may be changed, modified, edited, removed or withdrawn by MOHT at its absolute discretion and at any time without prior notice.

6.4 You shall obtain your own professional and/or legal advice relevant to your particular circumstances and conduct all necessary due diligence, including but not limited to making such investigations or seeking clarifications as may be appropriate, as regards any decision or action that you intend to take in relation to any matter concerning the mindline.sg web app. The mindline.sg web app does not cover all information available on a particular issue. The mindline.sg web app is not intended to be responsive to, inclusive of, or consistent with, any other sources of information available on any matters that may be addressed by or referred to by the mindline.sg web app.

6.5 The mindline.sg web app may contain hyperlinks to websites which are not maintained by MOHT. MOHT is not responsible for the contents or reliability of those websites and does not necessarily endorse the views expressed in them. MOHT has no control over and cannot guarantee the accessibility or proper functioning of those websites. Further, MOHT shall not be liable to you or any third party for any damage or loss whatsoever arising from your access to or use of those websites or third party information and materials. Use of the hyperlinks and access to those websites and materials are entirely at your own risk.

7. Security

7.1 MOHT, as appropriate, uses available technology for the protection of the security of any communications made via the mindline.sg web app . MOHT does not, however, accept any liability for the authenticity, confidentiality, integrity, and security of any communications and other transactions made through the mindline.sg web app. Internet communications may be susceptible to interception or interference by third parties.

8. Proprietary rights.

8.1 The materials contained in the mindline.sg web app, including all the information and software programmes are protected by copyright, trademark, and other forms of proprietary rights. All rights, title, and interest therein are licensed to and/owned and controlled by MOHT.

9. Data Protection Policy.

9.1 The full version of the Data Protection Policy applicable to the mindline.sg web app, can be viewed at a link on its main screen.

10. Governing Law

10.1 These Terms of Use shall be governed and construed in accordance with laws of the Republic of Singapore. Subject to paragraphs 10.2 and 10.3 below, any dispute arising out of or in connection with these Terms of Use, including any question regarding its existence, validity or termination, shall be referred to and finally resolved in the Courts of the Republic of Singapore and the parties hereby submit to the exclusive jurisdiction of the Courts of the Republic of Singapore. MOHT reserves the right to commence or maintain any proceedings for such dispute in the court of any country claiming or having jurisdiction in respect thereof and you irrevocably waive any objections you may have to such proceedings being brought before any such court.

10.2 MOHT may, at its sole discretion, refer any dispute referred to in paragraph 10.1 above to arbitration administered by the Singapore International Arbitration Centre (“SIAC”) in Singapore in accordance with the Arbitration Rules of the SIAC ("SIAC Rules") for the time being in force, which rules are deemed to be incorporated by reference in this clause. Further:

a. The seat of the arbitration shall be Singapore.

b. The tribunal shall consist of one (1) arbitrator.

c. The language of the arbitration shall be English.

10.3 Where MOHT is the defendant or respondent, it shall be given at least 30 days before the commencement of any legal action against it to elect to exercise the right herein to have the dispute submitted to arbitration. This right to elect shall not prejudice MOHT’s right to a limitation defence and the period to exercise the right shall not be abridged by reason of any accrual of a limitation defence in favour of MOHT during the said period.

### **DATA POLICY**

DATA PROTECTION POLICY ON THE MINDLINE.SG WEB APP

1. MOHT may use “cookies”, where a small data file is sent to your browser to store and track information about you when you enter our websites or use the mindline.sg web app. The cookie is used to track information such as the number of users and their frequency of use, profiles of users and their preferred sites. While this cookie can tell us when you enter our sites and which pages you visit, it cannot read data off your hard disk.

2. You can choose to accept or decline cookies. Most web browsers automatically accept cookies, but you can usually modify your browser setting to decline cookies if you prefer. This may prevent you from taking full advantage of the website.

3. For the avoidance of doubt, this policy is not intended to supersede or replace any consent previously provided to MOHT.

4. The mindline.sg web app does not ask for or collect data that identifies you. It may make use of your ‘user information’ comprising of nickname, age group, mood and topic(s) of interest. It is optional to enter this user information and you are instructed to not provide any information that would identify you. We may store this user information in the user’s end-user device in order to obtain continuity between sessions, but we will not transmit the this user information outside the device, or to our server. We do collect statistical data (“the Statistical Data”) as to which entries are selected so we can ascertain how anonymous users respond to the web app including analytics usage relating to links at this web app to third party websites, materials and/or services. Therefore this web app does not store any Personally Identifiable Information.

4.1 In line with MOHT's business operations, MOHT (including its related corporations, subsidiaries, affiliates, and associated companies) may collect and retain, as necessary and to the extent permitted by law, the Statistical Data, for effective delivery of services to the public and to facilitate its communications within the public and private healthcare sectors. The types of MOHT's business operations may include but may not necessarily be limited to:

- Day-to-day business and administrative operations of MOHT

- Research and services enhancement

- Corporate governance (including internal and company audits) and policy reviews

- Security and risk management

- Legal and regulatory requirements (including providing assistance to law enforcement, judicial and other government agencies)

- Administering and managing relevant relationships to conduct and further the business operations of MOHT

- Facilitating communications with individuals in connection with services provided by MOHT

- Any other purposes reasonably related to the above

4.2 We may share any Statistical Data collected with the Ministry of Health, the relevant regulatory authority for healthcare professionals, other statutory bodies and public agencies for the purpose of complying with their respective requirements, policies, and directives.

4.3 Any Statistical Data collected or provided to MOHT may also be shared among MOHT's appointed service providers performing data processing services to MOHT and authorised third party organisations providing and/or administering various MOHT programmes and schemes. This includes research partner organizations in Singapore and abroad.

4.4 To safeguard the Statistical Data collected, all electronic storage and transmission of Statistical Data is secured with appropriate security technologies.

4.5 MOHT will retain Statistical Data collected for as long as required for legal and business needs and requirements.

5. This site may contain links to third party websites, materials and/or services whose data protection and privacy practices may differ from ours. We are not responsible for the content and privacy practices of these other websites and encourage you to consult the privacy notices of those sites. If you click on such links, you will be directed to that third party's websites, materials and/or services. MOHT has no control over and assumes no responsibility for the content, privacy policies or practices of such third party websites, materials and/or services. MOHT shall not be liable to you or any third party for any damage or loss whatsoever arising from your access to or use of those third party websites, materials and/or services. Use of the links and access to those websites, materials and/or services are entirely at your own risk.

6. MOHT reserves the right to revise and update this data protection policy at any time. The content presented on this page is considered the latest version. Should any modification to this data protection policy occur, MOHT will post the updated version on the page in order that the visitors and users of this website are always made aware of how MOHT safeguards data.
